# The Low-Carbon Policy and Urban Green Total Factor Energy Efficiency: Evidence from a Spatial Difference-in-Difference Method

**DOI:** 10.3390/ijerph20043498

**Published:** 2023-02-16

**Authors:** Da Gao, Yanjun Cao, Chang Liu

**Affiliations:** 1 School of Law and Business, Wuhan Institute of Technology, Wuhan 430200, China; 2 School of Economics and Statistics, Guangzhou University, Guangzhou 510006, China; 3 School of Economics, Huazhong University of Science and Technology, Wuhan 430070, China

**Keywords:** low-carbon economy, energy efficiency, spatial spillover effect, mechanism analysis

## Abstract

In the post-epidemic background of the low-carbon economy and sustainable development, the low-carbon city pilot program (LCCP) is viewed as a practical method of improving energy efficiency. This study explores the spatial spillover effects of LCCP on green total factor energy efficiency (*GTFEE*) by developing a spatial difference-in-difference (SDID) model. Furthermore, we apply the mediating effects model to verify whether the rational allocation of resources is an influential channel for the spillover effect of LCCP policies. The results indicate that the LCCP policy has not only improved the local *GTFEE* by approximately 1.8%, but it also has a profound impact on the surrounding regions as well, which is about 76.5% that of the pilot cities. Additionally, the estimated results of the mediating effect model indicate that optimizing labor force and capital allocations are two essential channels through which the LCCP policy may contribute to improving regional cities’ *GTFEE*. Accordingly, the pilot cities should establish specific measures for rational resource allocation and promote the spatial spillover model of sustainable development.

## 1. Introduction

As the world’s largest source of energy consumption and carbon emissions [1], China’s industrial model and urbanization are characterized by high carbon emissions and energy consumption. Developing in such an unsustainable manner has resulted in several ecological and environmental issues, such as pollution and global warming, as well as a depletion of resources and energy [2,3,4]. The rising tension between resource availability and demand and ecological deterioration can be mitigated, some argue, by increased energy efficiency [5]. To fulfill carbon peaking and carbon neutrality commitments, China has launched a series of low-carbon policies, of which the low-carbon city pilot program (LCCP) has become a major innovation and advancement [6,7].

Aiming to implement and spread this new mode of transport, China’s National Development and Reform Commission (NDRC) has proposed three low-carbon city pilot initiatives covering all Chinese provinces in 2010, 2012, and 2017. Essentially, a low-carbon city is a city where the development model and direction are based on a low-carbon economy, citizens who live a low-carbon lifestyle, and governmental public management that uses the low-carbon society as a construction sample and blueprint. Characterized by lower energy consumption and pollution, this innovative urbanization strategy aimed to achieve the goal of a “Beautiful China” through cleaner production and green manufacturing.

Although the evaluation of low-carbon development and the effect of the LCCP program on green growth has aroused widespread attention [8,9], little research has examined the spatial spillover effects of LCCP on *GTFEE*, where local LCCP programs affect the *GTFEE* of surrounding areas in addition to those pilot sites. By optimizing resource allocation, technological innovation, and upgrading industrial structure, the LCCP aims to improve energy efficiency and total factor productivity in enterprises in the implemented cities [10,11]. A pilot city’s positive progress may be transferred to neighboring regions via knowledge spillover effects, leading to higher carbon productivity and *GTFEE* levels there. Nevertheless, the LCCP program can also lead to adverse spillover. The pilot cities’ strict standards and intense competitiveness will also cause companies with large emissions and immature technology to relocate to neighboring areas, which may decrease the *GTFEE* there. Thus, it is essential to examine the spatial spillover effects of LCCP to evaluate it accurately.

There are various mechanisms through which implementing the LCCP problem may improve *GTFEE*. First, the LCCP policy has significant positive effects on green total factor productivity (TFP), air quality improvement, and ecological efficiency in pilot cities [6,12], thus affecting *GTFEE* in the context of environmental aspects. Second, by optimizing resource allocation efficiency [13,14], LCCP policy will further affect carbon efficiency and green TFP in the context of economic aspects. Third, the R&D investment in green production, innovation in technology, and upgrading of the industrial structure brought about by the LCCP policy may affect the *GTFEE* of pilot cities in the context of technological aspects [10,11].

Accordingly, this study investigates the spatial spillover effects of the LCCP program by constructing a spatial difference-in-difference (SDID) model. Particularly, this paper first measures *GTFEE* in 277 Chinese cities from 2004 to 2019 with an undesirable slacks-based model (SBM), which is based on a total-factor framework. An SDID model is then constructed to estimate the spatial spillover effect of the LCCP program on *GTFEE*. Lastly, we apply a mediating effect model to examine whether misallocating resources contributes to spillover effects. This paper will address three questions: (i) Has the LCCP program successfully improved *GTFEE* in pilot cities? (ii) Does the LCCP program have any spatial spillover effects that may affect *GTFEE* within neighboring regions? (iii) How do those two phenomena occur?

Our research makes two primary contributions to the literature on the effects of the LCCP program and energy efficiency measurement. On the one hand, this paper contributes to the theoretical literature featuring LCCP policy evaluations by investigating the spatial spillover effect of the LCCP policy on energy efficiency. This study develops the SDID model derived from the spatial Durbin model in light of the following three considerations. Firstly, launching the LCCP policy in the pilot city may alter the spatial distribution of environmental status, technological development, and industrial structure, thus affecting the energy efficiency of surrounding regions. Second, the traditional and benchmark difference-in-difference (DID), which is used in most of the existing studies, requires that exogenous common shocks faced by both pilot cities and surrounding regions affect the pilot cities only and have no effect on other areas. Such a method may reduce the study’s accuracy, robustness, and credibility. Third, previous literature does not adequately address the spatial effects across regions and heterogeneity among the same region’s effects on other regions, thus biasing the results. Hence, we present SDID to evaluate the LCCP program’s impact on both the pilot cities and the surrounding areas of those cities.

On the other hand, this paper also fills the gap in mechanism analysis regarding how the LCCP policy affects the *GTFEE*. Most previous studies have examined only the effectiveness of the LCCP program without addressing how this program impacts *GTFEE*. This study constructs a mediating effect model to investigate the impact of the LCCP program on resource reallocation, demonstrating that the program mediates the resource mismatch phenomenon in implemented cities and neighboring regions, thereby supporting *GTFEE* improvement.

The remainder of this article is organized as follows: literature reviews are presented in Section 2. Section 3 outlines the methods and data. The discussion and empirical findings are detailed in Section 4. The robustness checks are reported in Section 5. Lastly, the conclusions and policy recommendations are summarized in Section 6.

## 2. Literature Review

### 2.1. The Studies Discussing the Impact of the LCCP Program on GTFEE

The greenhouse gases emission and the resulting global climate change have become common challenges for all mankind and imminent problems to be addressed immediately. Low-carbon cities, as a mode of sustainable growth, offer a viable solution to the conflicts between economic development, ecological protection, and resource conservation faced by traditional Chinese cities due to the dilemma of sustainable development [15]. Since the first batch of pilot low-carbon cities were initiated in 2010, many scholars have conducted in-depth studies on the impact of the policy. For example, Song et al. [16] confirm that the LCCP program improves urban ecological efficiency by using DID method. Qiu et al. [13] further affirm the role of LCCP in enhancing TFP and report the spatial heterogeneity of this role. Some scholars focus on the impact on energy efficiency. More specifically, Yu and Zhang [17] find that the LCCP program improves the pilot cities’ and neighboring areas’ carbon emission efficiency. Recently, Gao et al. [18] also confirmed the positive effects of LCCP policy on improving *GTFEE* and the spatial heterogeneity of the impact. Overall, the LCCP policy has played a substantial role in increasing energy efficiency and optimizing the ecological environment.

Most existing research, including the studies mentioned above, employ the DID model for policy evaluation of LCCP. Because spatial spillover effects are overlooked, such results may be biased [19,20]. The SDID model can address these deficiencies, which combines spatial econometric methods with the DID model [21,22]. The SDID method has been widely used in the impact measurement of several policies that are affected by spatial spillover effects [23], including policies related to high-tech industrial development [24], social welfare policy [25], and transportation infrastructure development [26,27]. However, few studies have considered spatial spillover while assessing the effects of LCCP policy on *GTFEE*.

### 2.2. The Existing Studies Regarding GTFEE

Many studies have focused on investigating the *GTFEE*. Most of the previous studies measure *GTFEE* under a single-factor framework by generating straightforward indices, including carbon emissions per *GDP* unit [28], carbon emission intensity [29], and energy intensity [30]. Although the above indices are concise and can represent energy efficiency to a certain extent, they are not a comprehensive reflection of energy efficiency because they are a relatively crude measure considering only a single factor [17,31]. This paper utilizes data envelopment analysis (DEA) for *GTFEE* analysis to measure energy efficiency based on the total factor framework. Although the full-factor perspective of measuring *GTFEE* solves some of the shortcomings of single-factor carbon emission efficiency, the traditional DEA does not contain undesirable outputs, or by default, all outputs are homogeneous and do not match realistic expectations. Hence, we apply the SBM model with undesirable outputs to measure the energy efficiency of Chinese cities.

### 2.3. The Influence Mechanism of the LCCP Program on GTFEE

In addition to studies related to the environmental and economic effects of LCCP policy, some scholars have continued to explore the influence mechanisms. According to Song et al. [16], the LCCP program improves the environmental sustainability of urban areas through technological innovation. Huang et al. [10] also affirm that applying this policy would encourage firms to invest more in research and development regarding sustainable manufacturing and promote innovation in the technology sector. In addition to technological innovation, decreasing the share of high-carbon industries induced by LCCP policy further promotes industrial structure upgrading [11]. Gao et al. [32] further investigate the influence mechanism through green innovation and structural upgrading paths. However, most of this research has analyzed the mechanisms of LCCP policy based on technological innovation and industrial structure while ignoring the possible effects of the policy on improving the misallocation of resources. Whereas Chen et al. (2021) analyze LCCP policy effects on factor productivity in the context of optimizing resource allocation efficiency, this study focuses on the impact on *GTFEE* considering influence mechanisms of mitigating resource misallocation of labor and capital.

Improving resource misallocation is indeed one of the important paths for the LCCP policy to impact *GTFEE*. Initially, this program can foster environmentally sustainable innovation by optimizing resource allocation and mitigating financing constraints [1]. Considering its substantial upfront investment, prolonged cycle time, and unpredictable risks, enterprises need firmer support from the capital and labor market to alleviate the financing constraints they face in the green innovation process compared to traditional innovation [33,34,35]. At present, the LCCP program provides a variety of economic incentives to drive funding flow to green production. These include establishing low-carbon and green innovation funds, the provision of loan subsidies, and providing cash incentives. In this way, capital and labor resources flow to green industries and innovations and thus may improve the *GTFEE* by optimizing resource allocation.

In summary, most existing studies have neglected the spatial spillover effects of the LCCP program, making it difficult to obtain an unbiased and comprehensive estimate of the LCCP policy’s impact on *GTFEE*. Moreover, previous literature rarely investigates the mechanism of LCCP policy, especially through the resource allocation aspect. To fill the gaps, this study constructed the SDID method to analyze the spatial spillover of the LCCP policy on *GTFEE*. Furthermore, a mediating effect model is also applied to examine how the LCCP program produces such effects.

## 3. Models and Data

In this section, to illustrate the extent to which the LCCP policy affects the *GTFEE* and how such impacts generate, the SDID method and the mechanism analysis are employed to investigate the spatial spillover effect of the LCCP program on *GTFEE*.

### 3.1. The SDID Model

Referring to Dai et al. [20], this study constructs an SDID model that is derived from the Stochastic Impacts by Regression on Population, Affluence, and Technology model (STIRPAT). The STIRPAT model has been frequently utilized to examine environmental influences on socioeconomic factors [36] and to identify factors that affect the indicators associated with the environment [37,38]. A traditional STIRPAT model to analyze the determinants of energy efficiency is as follows:

(1) $$GTFEE_{it}=\alpha_{0}+\alpha_{1}lnDFD_{it}+\alpha_{2}lnPD_{it}+\alpha_{3}lnEDL_{it}+\alpha_{4}lnIC_{it}+\alpha_{5}GC_{it}+\lambda_{t}+\mu_{i}+\varepsilon_{it}$$

where the calculated *GTFEE* is the dependent variable. The independent variables include the degree of fiscal decentralization (*DFD*), population density (*PD*), economic development level (*EDL*), infrastructure construction (*IC*), and green coverage in constructed areas (GC) [17,32]. The subscripts *t* and *i* designate the corresponding time period and city, respectively. The natural logarithm is indicated by *ln*.

To investigate the impact of the LCCP program, various scholars have developed the previous STIRPAT model into a DID model. Nevertheless, DID can only assess the effects of the LCCP policy on specific cities and cannot estimate the spatial spillover effects on surrounding regions. In this regard, such estimation becomes less reliable and accurate. Similar to the methods in Dai et al. [20], this study applies the SDID model to evaluate the comprehensive effects of this program on the *GTFEE* by incorporating both the direct effects on pilot cities and the spillover effects on neighboring areas. The corresponding SDID model is formulated as follows:

(2) $$GTFEE_{it}=\alpha_{0}+\rho WGTFEE_{it}+\delta_{1}Did_{it}+\delta_{2}WDid_{it}+\beta_{i}\sum_{i}^{k}X_{i}+\gamma_{i}W\sum_{i}^{k}X_{i}+\lambda_{t}+\mu_{i}+\varepsilon_{it}$$

where $GTFEE_{it}$ is the *GTFEE* of sample city *i* in year *t*, and $X_{i}$ represents the *i*th control variable with the total number of *k*. $Did_{it}$ is the dummy variable to specify whether city *i* has implemented the low-carbon policy in year *t*. *W* is the spatial weights matrix.

There are four common spatial matrices according to different distance measurement methods, which are the economic geographic nested matrix, spatial geographic distance matrix, economic distance matrix, and adjacency matrix. We apply the nested matrix, by which elements account for the geographical and economic distances between two cities, to examine the spatial correlation of *GTFEE*. Compared with the other three, such distance measurement methods can more comprehensively measure the gap between cities. The interaction term $WDid_{it}$ represents how influential is the LCCP policy of the pilot city on the *GTFEE* of neighboring regions; $WGTFEE_{it}$ indicates the spatial lag term of *GTFEE*, more specifically, the influencing degree of the *GTFEE* of the assigned pilot city on that of the surrounding areas. $\lambda_{t}$ denotes time-fixed effects, $\mu_{i}$ denotes city-fixed effects and $\varepsilon_{it}$ is the stochastic error term.

The spatial autoregressive model (SAR), spatial error regression model (SEM), and spatial Durbin model (SDM) are three widely applied models in SDID methods. More specifically, the SDM can be considered as the SAR when $r_{i}=0$ and as SEM when $\beta_{i}r_{i}+\rho =0$. To make the estimation results more comprehensive, our subsequent tables report the estimated parameters under all three models simultaneously.

### 3.2. The Mediating Effect Model

The last section constructs the SDID model to investigate the spatial spillover effects of the LCCP program on *GTFEE*. Meanwhile, scholars are also uncertain about how the program affects the *GTFEE* in the surrounding regions. Several studies have demonstrated that the LCCP policy optimizes resource allocation [1,4]. Then, the LCCP program may affect *GTFEE* in the surrounding areas in two ways. One is that the awareness of the government’s strong support for green production may spread to the surrounding areas, thus will guide the capital and labor resource. In addition to driving green production in the pilot city, the LCCP policy may also motivate enterprises in surrounding regions to do the same. We propose that the reallocation of the labor force and the capital resources may serve as two mediating variables to explain the spatial spillover effects of the LCCP policy on the *GTFEE* in nearby areas.

Based on the basic model, we further develop the following two mediating effects models, combined with the above Equation (2), to analyze the mediating effect of the low-carbon policy on *GTFEE*.

(3) $$\;\;\;\;\;\;M_{it}=\alpha_{0}^{\prime }+\rho^{\prime }WGTFEE_{it}+\delta_{1}^{\prime }Did_{it}+\delta_{2}^{\prime }WDid_{it}+\beta_{i}^{\prime }\sum_{i}^{5}X_{i}+\gamma_{i}^{\prime }W\sum_{i}^{5}X_{i}+\lambda_{t}^{\prime }+\mu_{i}^{\prime }+\varepsilon_{it}^{\prime }$$

(4) $$GTFEE_{it}=\alpha_{0}+\rho WGTFEE_{it}+\delta_{1}Did_{it}+\delta_{2}WDid_{it}+\beta_{i}\sum_{i}^{5}X_{i}+\beta_{6}M_{it}+\gamma_{i}W\sum_{i}^{5}X_{i}+\gamma_{6}WTL_{it}+\;\lambda_{t}^{\prime \prime }+\mu_{i}^{\prime \prime }+\varepsilon_{it}^{\prime \prime }$$

where $M_{it}$ represents the mediating variable, which denotes the labor misallocation index ($TL_{it}$) and the capital misallocation index ($TK_{it}$) for two estimations. We estimate the Equations (2)–(4) two times by choosing the $TL_{it}$ and $TK_{it}$ as $M_{it}$ to investigate the mediating effects of labor misallocation and capital misallocation, respectively.

**Figure 1:**
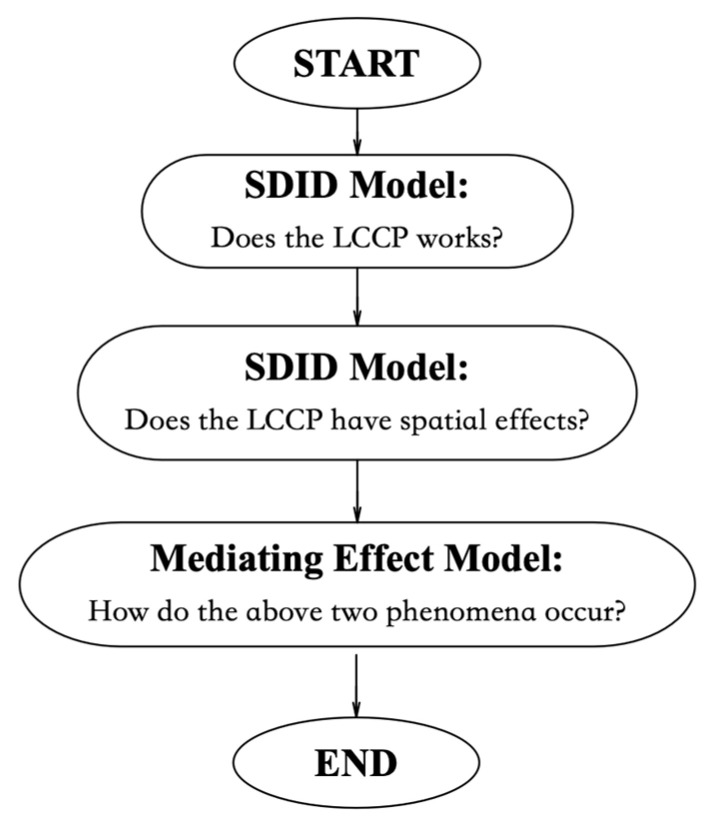
Modeling Steps.

To clarify our empirical model more directly, we present the modeling steps in Figure 1 to analyze the three questions we bring out in the introduction section: (i) Has the LCCP program successfully improved *GTFEE* in pilot cities? (ii) Does the LCCP 76 program have any spatial spillover effects that may affect *GTFEE* within neighboring regions? (iii) How do those two phenomena occur?

### 3.3. Variables

In this section, we introduce the dependent, independent, mediating, and control variables. Definitions for variables are shown in Table 1.

#### 3.3.1. Dependent Variable

The dependent variable of this paper is the measured *GTFEE*. Before constructing the SDID model, this study first needs to measure the *GTFEE* to be the indicator as the dependent variable. Referring to Gao et al. [39], we utilize the undesirable-SBM model to assess the *GTFEE* of 277 Chinese cities from 2004 to 2019. More specifically, this study assumes that each of the N decision-making units (DMU) has M inputs, $s_{1}$ expected outputs, and $s_{2}$ undesirable outputs, respectively, which can be expressed in the form of matrix $X=\left(x_{ij}\right)\in R_{m\times n}$, $Y^{g}=\left(y_{ij}^{g}\right)\in R_{s_{1}\times n}$, $Y^{b}=\left(y_{ij}^{b}\right)\in R_{s_{2}\times n}$; the relaxation vectors of inputs are $s^{-}\in R_{m}$; the desirable outputs and undesirable outputs are $s^{g}\in R_{s_{1}}$ and $s^{b}\in R_{s_{2}}$, and λ stands for the weight vector. *GTFEE* can be measured by the following formula:

$$\min\;p=\frac{1-\left(1/m\right)\sum_{i=1}^{m}s_{i}^{-}/x_{i0}}{1+\frac{1}{s_{1}+s_{2}}\left(\sum_{r=1}^{s_{1}}s_{r}^{g}/y_{r0}^{g}+\sum_{r=1}^{s_{2}}s_{r}^{b}/y_{r0}^{b}\right)}$$

(5) $$s.t.\{\begin{array}{c} x_{0}=X\lambda +s^{-}\\ y_{0}^{g}=Y^{g}\lambda -s^{g}\\ y_{0}^{b}=Y^{b}\lambda -s^{b}\\ \lambda \geq 0,\;s^{-}\geq 0,\;s^{g}\geq 0,\;s^{b}\geq 0 \end{array}$$

The capital resource, the labor force, and energy consumption are included in the input. The desirable output is *GDP*, whereas the undesirable output includes ${\mathrm{SO}}_{2}$, smoke, and effluents [40,41]. Notably, we can solve Equation (5) using the linear programming solver on MATLAB2018a software.

#### 3.3.2. Independent Variable

A series of low-carbon pilots were initiated in 2010, including Guangdong, Hubei, Shanxi, Yunnan, Liaoning provinces, and 82 other cities. After that, the 2nd and 3rd batches of pilots contain 28 and 45 cities (counties and districts), respectively. The detailed lists of pilots are presented in Table 2. Dummy variable LCCP policy, $Did_{it}$, which denotes the implementation of LCCP policy, is the independent variable. If the LCCP program has been implemented in city *i* at period *t*, then $Did_{it}$ equals 1; otherwise, it equals 0.

#### 3.3.3. Mediating Variables

The mediating variables in this paper are the mismatch index of the capital resource and labor force. Labor mismatch is a kind of factor mismatch that mainly refers to the situation in which the Pareto-optimal labor allocation cannot be achieved due to insufficient labor mobility. Thus, there is a certain loss in production efficiency and total output. Generally speaking, labor mismatch arises when the marginal substitution rate of labor factors in a certain industry or region differs from that of other industries or regions. Capital mismatch refers to the distortion phenomenon that capital, a scarce resource, defies the laws of the market economy under the action of some human factors and flows excessively to certain regions, industries, and enterprises with low economic efficiency, while the capital needs of other regions, industries, and enterprises with relatively high economic efficiency are not effectively met.

Based on the method in Bai and Liu [42], we measure the labor force mismatch index $TL_{it}$ and capital resource mismatch index $TK_{it}$ to represent the resource misallocation. As shown in Equation (6) and Equation (7), the $\zeta_{Li}$ and $\zeta_{Ki}$ are the distortion coefficients of the labor force and capital resource, respectively. Equations (8) and (9) illustrate the price relative distortion coefficients employed in the actual measurement. More specifically, $L_{i}$ and $K_{i}$ denote the actual used resource of labor and capital; L and K represent the total resource of labor and capital; $S_{i}$ represents the proportion of the output of city *i* to the total output; $\eta_{Li}$ and $\eta_{Ki}$ denote the output elasticity of labor resource and capital resources that are calculated by the solo residual method; and $\eta_{L}=\overset{N}{\underset{i}{\sum }}S_{i}\eta_{Li}$ and $\eta_{K}=\overset{N}{\underset{i}{\sum }}S_{i}\eta_{Ki}$ generate the output-weighted value of labor and capital contribution. Hence, $\frac{L_{i}}{L}$ and $\frac{K_{i}}{K}$ represent the ratio of resource utilization, which are the actual used resource to the total resource in the city *i*; $\frac{S_{i}\eta_{Li}}{\eta_{L}}$ and $\frac{S_{i}\eta_{Ki}}{\eta_{K}}$ represent the theoretical proportion of the labor force used in city *i* when the labor force is efficiently allocated. In this way, further deviations of the index from zero indicate more severe resource misallocation. Thus, the absolute value of the index is regarded as an indicator of resource misallocation.

(6) $$TL_{it}=\frac{1}{\zeta_{Li}}-1$$

(7) $$TK_{it}=\frac{1}{\zeta_{Ki}}-1$$

(8) $$\hat{\zeta_{Li}}=\frac{\frac{L_{i}}{L}}{\frac{S_{i}\eta_{Li}}{\eta_{L}}\;}$$

(9) $$\hat{\zeta_{Ki}}=\frac{\frac{K_{i}}{K}}{\frac{S_{i}\eta_{Ki}}{\eta_{K}}\;}$$

#### 3.3.4. Control Variables

In this paper, the following variables are selected as the control variables to explore the relationship between low-carbon policy and urban *GTFEE* by referring to the existing relevant literature on influencing urban *GTFEE*. To be specific, we control the degree of fiscal decentralization (*DFD*), population density (*PD*), economic development level (*EDL*), infrastructure construction (*IC*), and green coverage in constructed areas (*GC*). Referring to Gao et al. [18], we argue that *DFD* and *EDL* may impact the *GTFEE* of cities, that fiscal decentralization is beneficial to cities’ self-management, and that the level of economic development has an impact on their green development, thus improving energy efficiency. *DFD* is calculated from the proportion of public revenue to public expenditure. In the calculation of *PD*, the total population of an administrative division is divided by the area of that division. With reference to Liu et al. [42], *PD* and *IC* are considered important indicators of urbanization development and, in our opinion, impact the green development sustainability of cities. *EDL* is generated from the average *GDP*; *IC* is measured in road distance per inhabitant. Referring to Cheng et al. [15], the green coverage of the city contributes to the absorption of CO_2_, which may have an impact on the measurement of the energy efficiency of the city. *GC* indicates the green coverage in the constructed area.

### 3.4. Data

This study selects 277 Chinese cities during 2004–2019 as samples to analyze the spatial spillover effect of the LCCP program on *GTFEE*. Information regarding the program can be obtained from the National Development and Reform Commission (NDRC). Data used for the calculation of *GTFEE* and the other mechanism and control variables are derived from the China City Statistical Yearbook and China Energy Statistical Yearbook. The descriptive statistics are presented in Table 3.

## 4. Empirical Results

This section first discusses the results of the parallel trend test. Then the spatial spillover effects of the LCCP program on *GTFEE* are reported. Finally, the influencing mechanisms of the policy are analyzed. Notably, the results from this section’s series of empirical analyses are calculated using STATA17 software.

### 4.1. Parallel Trend Test and Dynamic Effects Analysis

A crucial assumption for applying the SDID model is that the *GTFEE* of cities with or without the policy implementation had the same developing trend before the policy had been applied. Hence, to verify that this prerequisite has been met, this study conducts a parallel trend test. Following Lin [43] and Yin and Guo [44], this study utilizes the event research method to test the parallelism of the trend across multiple time points. The corresponding equation is as follows:

(10) $$GTFEE_{it}=\alpha_{0}^{\prime \prime \prime }+\rho^{\prime \prime \prime }WGTFEE_{it}+\sum_{-5}^{5}\delta_{k}Did_{it}^{k}+\delta_{6}WDid_{it}+\beta_{i}^{\prime \prime \prime }\sum_{i}^{k}X_{i}+\gamma_{i}^{\prime \prime \prime }W\sum_{i}^{k}X_{i}+\mu_{i}^{\prime \prime \prime }+\lambda_{t}^{\prime \prime \prime }+\varepsilon_{it}^{\prime \prime \prime }$$

where $Did_{it}^{k}$ is the dummy variable indicating the implementation of LCCP policy, and other variables are the same as those mentioned above. We use 5 as the threshold for setting the dummy variable. Assuming that $Y_{i}$ is the start year for the LCCP program, $Did_{it}^{k}$ indicates $t-Y_{i}=k$, k∈[−5, 5]. Since $\delta_{k}$ represents the policy effects on *GTFEE*, $\delta_{-5}-\delta_{-1}$ can test the parallel trend [45]. In other words, only if the direct effect of $Did_{it}^{-5}-Did_{it}^{-1}$ is not statistically significant, will the parallel trend test be passed.

**Figure 2:**
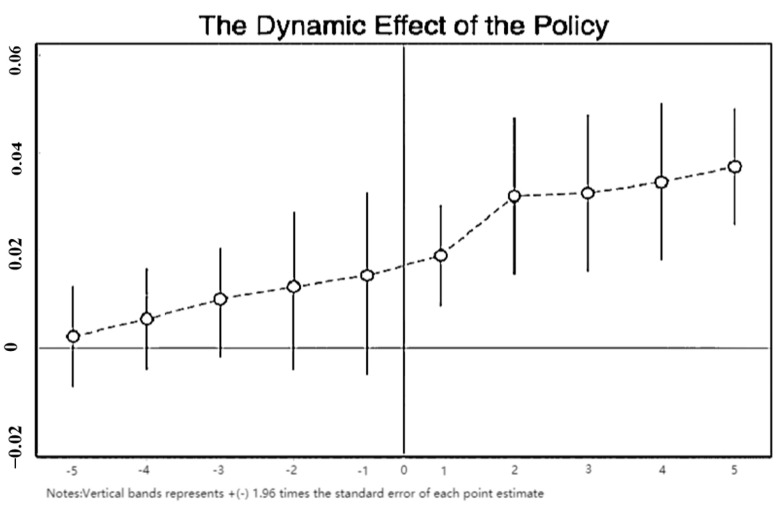
Parallel Trent test and the dynamic effect analysis of the LCCP Policy.

Using the geographic distance matrix, Figure 2 presents the direct effects and 95% confidence interval of $Did_{it}^{k}$. The horizontal axis is the 5 years before and after the launching year of the LCCP program, denoted as d_5 to d5. The red line represents the benchmark year when the policy was introduced, which is deleted as a base period. As shown in Figure 2, no significant alteration has been observed between the treatment and control groups. Hence, the assumption of parallel trends in *GTFEE* in the pilot and nonpilot cities before the benchmark year of the LCCP program has been satisfied. As for the dynamic effect of the LCCP policy, *GTFEE* showed significant improvement after the implementation of the pilot policy, and the policy effects were evident in the following years. This is one of the arguments for the effectiveness of the policy.

### 4.2. Spatial Spillover Effects of the LCCP Policy

Table 4 reports the benchmark regression results estimated through Equation (2), in which Model (1) is estimated by the SAR method without the control variables and Model (2)–(4) are estimated by the SAR, SEM, and SDM methods with all control variables. The spatial weight matrix W is generated by the geographic distance.

Comparing Model (1) and Model (2), it is obvious that LCCP policy has a significant positive impact on *GTFEE*, whether controls are included or not. As shown in Model (2)–(4), the three estimated coefficients of variable *Did* based on different models are all positive and statistically significant, which means implementing the LCCP policy significantly improved the *GTFEE*. Our findings are consistent with existing studies and confirm the positive role of LCCP policy on the environmental and economic aspects [15,43,46].

The estimated policy effects obtained in this study range from 0.012 to 0.018. Although both confirm the positive effects of the LCCP program on improving *GTFEE*, the results are slightly smaller than the valuation interval of 0.036–0.038 obtained by Gao et al. [18]. Compared to this study, the coefficients estimated by the previous study through the multiple DID analysis, without considering the spatial spillover effects, may overestimate the policy effect of the LCCP. Moreover, the estimated coefficients of *Did* in Gao et al. [18] are positive at 5% significance levels, while our results are at 1% significance levels. Hence, this SDID estimation model, which integrates spatial spillover effects, would provide a more accurate and robust estimation of LCCP policy effects coefficients.

According to the results, besides the positive estimation of *Did*, the spatial lag terms of *GTFEE* in Model (2) and (4), as well as the disturbance term in Model (3), are also positive and statistically significant with *p* < 0.01. In summary, these estimates indicate that the LCCP program has a positive spatial spillover effect. All these three models have confirmed such positive effects. Other than that, the estimated coefficient of *WDid* is positive at a 5% significant level in Model (4), which means the implementation of the LCCP program in the surrounding areas also has positive effects on improving the city *i*’s *GTFEE*. In general, there are two aspects of the positive spatial spillover effects, which are the effects of implementation policy in the pilot city and improving *GTFEE* in the pilot city. These two sources of the spatial spillover effects both have a positive contribution to improving the *GTFEE* in surrounding regions.

Moreover, some covariables also have strong explanatory power. Among those factors, infrastructure construction (*lnIC*) is negative at a 1% significance level for all three models. Until new energy is fully utilized in each vehicle on the road, the greater the road miles per capita, i.e., the stronger the infrastructure development, tends to increase total carbon emissions and reduce energy efficiency. The estimated results for *lnPD* are also significantly positive for the three models, demonstrating that population growth has contributed to improvements in energy efficiency. The coefficients of *lnEDL* are negative but not statistically significant, implying that economic growth’s positive effects on improving *GTFEE* may not materialize shortly.

### 4.3. Mechanisms Analysis

The last section shows the significant positive effect of the LCCP policy on *GTFEE*. Then, what are the influencing mechanisms behind the phenomenon? To answer the question, we employ the mediating effect model through the SDM method to identify whether resource misallocation can act as a mediator for the estimated spatial spillover effects. The results of two mediating variables (misallocation in labor and capital) are presented in Model (5)–(6) and Model (7)–(8) of Table 5.

As for testing the mediating effect of labor misallocation, Model (6) reports the results estimated by Equation (4) and Model (7) reports estimation results from Equation (3). As shown in Model (5), the estimated coefficient of *Did* is negative at the 1% significance level, which indicates that the implementation of the LCCP program has a significant role in optimizing the allocation of labor resources. Model (6), which are the estimated results of Equation (4), shows that resource misallocation has a notable adverse effect on improving *GTFEE*, which could indicate a mediating factor.

Moreover, the coefficient of the spatial lag term of the LCCP policy, which is the *WDid*, in Model (6) is statistically significant, meaning that labor misallocation has an incomplete mediating effect. Similar to the discussion about labor misallocation, Model (7)–(8) are the results for analyzing the mediating effect of capital misallocation. As shown in Model (7), the estimated coefficient of *Did* is also negative at the 1% significance level, which means that the implementation of the LCCP program has a significant role in optimizing the allocation of capital resources. Combined with the results in Model (8), it is indicated that capital misallocation has a complete mediating effect.

## 5. Robustness Check

In this section, several alternative specifications are discussed to test the robustness and adequacy of our findings. The results in this study are impressively robust to the following checks.

### 5.1. PSM-SDID Model

First, this study incorporates the SDID model with the propensity score matching method (PSM-SDID) to excavate a fitting control group for the treatment group. The results of the matching balance test of each matching characteristic variable are presented in Table 6. The matching procedure reduced the bias of all variables. It should also be noted that the *t*-statistic does not indicate significance, which suggests that the matching has met the balance requirement.

Table 7 presents the regression estimation results of the PSM-SDID model, including all three methods (SAR, SEM, and SDM). In general, the results are similar to our empirical model estimation, and the estimated coefficients of *Did* are positive at a 1% significance level. The spatial lag terms of *GTFEE* (estimated coefficient ρ) are positive and statistically significant with *p <* 0.01 in Model (9) and (11), and the disturbance term is also significantly positive in Model (10). This means that the boosting effects and positive spillover effects of the LCCP program on improving *GTFEE* are still significant and stable.

### 5.2. Spatial Weights Matrix Selection

Second, the selection of the spatial weights matrix is an important factor that may influence the estimation of spatial spillover effects. Hence, in the robustness check, the original nested matrix W has been replaced with the adjacency matrix W1, the economic distance matrix W2, and the spatial geographic distance matrix W3, by which elements account for the geographical distances between two cities. These results are presented in Table 8. Models (12)–(14) are estimated results through the SDM method with different spatial weights matrices. As shown in Table 8, with the new spatial weights matrices, the SDM method is still applicable. Furthermore, the estimated coefficients of *Did* and its spatial lag term are still significantly positive in the following three models. These findings are consistent with the original empirical estimation that the LCCP policy has a positive spatial spillover effect on improving *GTFEE*, and the results obtained before are relatively robust.

### 5.3. Replacing the Measure of the Explanatory Variable

Since different estimation methods have significant differences in the estimation results of *GTFEE*, we refer to Gao et al. [5] and use the Epsilon-based (EBM) model to measure the city *GTFEE*, which integrates radial and non-radial features. It has the advantage of being more accurate and comprehensive in efficiency evaluation. Therefore, the EBM model is used to calculate cities’ green total factor energy efficiency as the explained variable. We also use the SDID model above for the robustness test. These results are shown in Table 9 in Models (15)–(17) for the SAR, SEM, and SDM methods using the Epsilon-based measure (EBM) model to measure the *GTFEE* of the city. Overall, the results are similar to our baseline model estimates, with the estimated coefficients of *Did* being positive at the 1% significance level and the spatial lags of *GTFEE* (estimated coefficients ρ) being positive and statistically significant (*p* < 0.05). This indicates that the results of the positive spatial spillover effect of the LCCP policy on increasing *GTFEE* are relatively robust.

## 6. Heterogeneity Analysis

China is a vast country, and many factors, such as geographical characteristics and economic development status, vary greatly between regions. Therefore, we further investigated the heterogeneity of LCCP in influencing urban *GTFEE* according to the geographical location and comprehensive economic development of Chinese prefecture-level cities.

### 6.1. The Heterogeneity of Urban Economic Development Levels

Since the degree of marketization, industrialization, and autonomy varies greatly depending on the level of urban development, we have considered five categories based on the criteria for classifying cities published by the China Financial Business Journal in 2018, namely: commercial resources, urban centers, population size, cultural diversity, and urban potential. We classify cities into five levels, the first, second, third, fourth, and fifth-tier cities, based on the above conditions, to study the heterogeneous effect of LCCP policies on improving *GTFEE*.

Model (18)–(22) in Table 10 report the results estimated from the highest level of development to the lowest level of development, respectively. The extent to which LCCP policy effectively improves energy efficiency is more pronounced in cities with higher levels of economic development. For the first and second subgroups, the estimated coefficients are significantly positive, and the coefficients’ magnitudes are also higher than the original sample and the subgroups of lower development levels. In addition to the direct effect on *GTFEE*, as shown in the estimated coefficients of ρ, the positive spatial spillover effects of the LCCP program are more pronounced in cities with higher levels of economic development. While the ρ estimations of five subgroups are all positive, suggesting that LCCP policy has positive spatial spillover effects regardless of the economic status of cities, the magnitudes of estimations are lowered as the level of urban development decreases.

The reason behind this is the phenomenon of economic agglomeration; the more developed the cities, the higher the degree of economic agglomeration, which makes it easier for the green development achieved by the city. At the same time, we believe that economic agglomeration may positively impact *GTFEE* in two ways. First, lowering the output consumption per unit of production factor may reduce the industry’s average cost. Second, economic agglomeration may promote technological and industrial upgrading through knowledge spillover effects and peer pressure (Fujita et al., 1999).

### 6.2. The Heterogeneity of Urban Geographic Locations

In order to effectively identify the heterogeneous effects of regional differences on the urban *GTFEE* of LCCP programs, based on the previous empirical study, the empirical study was further divided into regions to examine the LCCP programs under the three major regions of East, Central, and West. The multi-period SDID model was still used for estimation, and the estimation results are shown in Table 11. Model (23)–(25) shows that the implementation of the LCCP programs has a significant impact on the *GTFEE* of the eastern and central cities. There is a significant spatial spillover effect, which indicates that the eastern central city plays a radiative effect on the city and contributes significantly to the *GTFEE* of the surrounding cities.

This may be because the cities in the eastern and central regions are economically developed. Thus, when the LCCP policy is implemented, there is an obvious promotion for the energy efficiency of the cities in regions, where the energy efficiency of the cities with leading positions in the region is significantly higher than that of other cities, and at this time the neighboring cities will benefit under the spatial spillover effect. We also find that the promotion effect of *GTFEE* is also higher in the eastern cities than in the central cities. For western cities, implementing the LCCP programs has no significant impact. The reason may be that the cities in the western region are relatively backward, and the implementation of the LCCP policy significantly affects the cities’ energy efficiency. Although there is a certain connection between the *GTFEE* between cities, this spatial spillover effect is not significant because the energy efficiency of the cities in the western region is relatively low.

## 7. Conclusions

This study discusses the policy effects and the spatial spillover effects of the LCCP program on China’s urban *GTFEE* from 2004 to 2019. Generally, we first analyze the spatial correlation of *GTFEE* through the undesirable-SBM model. Then, the SDID model was developed to investigate the spatial spillover effects of LCCP policy on improving *GTFEE*. Finally, to discuss the occurrence mechanism of the policy effects and the spatial spillover effect, this study accurately measured the mediating effects of labor and capital misallocation in the impact of the LCCP program on *GTFEE*. The analysis suggests that the LCCP program has significant positive policy effects and spatial spillover effects on improving *GTFEE*. Furthermore, optimizing labor and capital allocation is an intermediary influencing factor of such effects.

In general, we find out following answers to the three questions we had asked before: (i) The implementation of the LCCP policy significantly improved the *GTFEE* in the pilot city by 1.8%. Compared with previous effect measures for the LCCP program, this paper takes into account the spatial spillover effects of the LCCP program, avoids possible overestimation of LCCP program effects, and obtains more accurate and better statistically significant estimation results. (ii) The LCCP program has positive spatial spillover effects that may affect *GTFEE* within neighboring regions, which specifically comes from two aspects: first, the implementation of LCCP policy in the pilot city has a profound impact on the *GTFEE* of the surrounding regions, which is about 76.5% of that on the *GTFEE* of the pilot cities. Second, the impact of improved *GTFEE* in the pilot city on the enhancement of *GTFEE* in the surrounding areas. These two sources of the spatial spillover effects both have a positive contribution to improving the *GTFEE* in surrounding regions. (iii) The implementation of the LCCP program has a significant role in optimizing the allocation of labor and capital resources. In contrast, the labor and capital resource misallocation has notable adverse effects on improving *GTFEE*, which could indicate that these two factors have mediating effects. The direct impacts and spatial spillover effects of the LCCP program on *GTFEE* may occur through the reallocation of labor and capital resource. There are some endogeneity problems in the mediation effect model. Although the mediation effect model in this paper can accurately reveal the influence of technological innovation and resource mismatching on technological innovation efficiency, it is not perfect, which is one of the limitations of this paper. Therefore, exploring the mediation effect model to avoid endogeneity is also one of the future research directions of this paper.

According to those findings, we put forward the following three policy implications. Firstly, it can be seen that the LCCP policy has a positive effect on improving energy efficiency. Therefore the policy should be more strongly supported and promoted in more regions. Considering the effectiveness of the LCCP program, the government should increase its efforts to implement policies related to the project, such as promoting energy saving and emission reduction in heavy industries, optimizing energy consumption structure, improving various waste treatment, controlling oil and grease and aversive emissions, etc., in order to build a low-carbon economy and optimize the low-carbon environment in a targeted manner.

Secondly, empirical results show that LCCP policy has a positive spatial spillover effect on urban *GTFEE*. Therefore, when implementing the LCCP policy in more developed cities, the Chinese government and relevant policymakers should use the radiation effect fully. Implementing and supervising the policy can not only increase the incentive effect on the green development of enterprises in the implementation area. It also promotes energy efficiency and green production technology in surrounding cities and better radiates the surrounding areas.

Thirdly, the empirical results of the mechanism analysis in this paper can help the government make more efforts to promote the rationalization of resources. LCCP policy has the obvious effect of reducing the mismatch of labor and capital. The rapid flow of capital will be conducive to cities’ capital flow and knowledge spillover. At the same time, with the more rational allocation of labor resources, it is more conducive to play the advantages of the agglomeration economy and improve the energy efficiency of cities.

## Figures and Tables

**Table 1:** Variables and definitions.

Variable	Definitions
Dependent Variable:	
*GTFEE*	Measured green total factor energy efficiency
Independent Variable:	
*Did*	Denotes the implementation of the Low Carbon City Pilot program
Mediating Variable:	
*TL*	Labor force mismatch index
*TK*	Capital resource mismatch index
Control Variables:	
*DFD*	Degree of fiscal decentralization
*PD*	Population density
*EDL*	Economic development level
*IC*	Infrastructure construction
*GC*	Green coverage in constructed areas

**Table 2:** List of Pilots from 2010 to 2017.

Batches	Regions (Including Provinces, Cities, Districts, and Counties)
First (2010)	Guangdong, Liaoning, Hubei, Shanxi, Yunnan; Tianjin, Chongqing, Shenzhen, Xiamen, Hangzhou, Nanchang, Guiyang, Baoding
Second (2013)	Hainan, Beijing, Shanghai, Shijiazhuang, Qinhuangdao, Jincheng, Hulun Buir, Jilin, Greater Hinggan Mountains region, Suzhou, Huai’an, Zhenjiang, Ningbo, Wenzhou, Chizhou, Nanping, Jingdezhen, Ganzhou, Qingdao, Jiyuan, Wuhan, Guangzhou, Guilin, Guangyuan, Zunyi, Kunming, Yan’an, Jinchang, Urumqi
Third (2017)	Nanjing, Changzhou, Jiaxing, Jinhua, Chuzhou, Sanming, Jinan, Yantai, Weifang, Zhongshan, Shenyang, Dalian, Chaoyang, Xunke, Sanya, QiongZhong, Hefei, Huaibei, Huangshan, Liuan, Xuancheng, Gongqingcheng, Gian, Fuzhou, Changyang, Changsha, Zhuzhou, Xiangtan, Chenzhou, Wuhai, Liuzhou, Chengdu, Yuxi, Puer, Lhasa, Ankang, Lanzhou, Dunhuang, Xining, Yinchuan, Wuzhong, Changji, Yining, Hotan, Xinjiang Corps

**Table 3:** Summary statistics.

Variables	N	Mean	SD	Min	Max
*GTFEE*	4048	0.286	0.0957	0.106	1
*Did*	4048	0.175	0.380	0	1
*lnIC*	4048	3.144	0.541	0.468	5.207
*lnEDL*	4048	10.19	0.807	4.595	13.06
*GC*	4048	37.74	13.72	0	386.6
*lnDFD*	4048	−0.811	0.485	−3.664	0.433
*lnPD*	4048	5.844	0.776	2.872	7.887
*TK*	4048	0.006	0.431	−0..984	5.112
*TL*	4048	−0.002	0.415	−0.918	2.442

**Table 4:** Benchmark regression estimation results.

Variables	(1)	(2)	(3)	(4)
	SAR	SAR	SEM	SDM
*Did*	0.009 ***	0.012 ***	0.018 ***	0.018 ***
	(3.75)	(4.40)	(5.22)	(4.85)
*lnDFD*		0.013 ***	0.008 *	0.006
		(3.41)	(1.79)	(1.22)
*lnPD*		0.025 ***	0.018 ***	0.015 **
		(4.20)	(2.86)	(2.44)
*lnEDL*		0.000	−0.006	−0.012 **
		(0.21)	(−1.49)	(−2.56)
*lnIC*		−0.010 ***	−0.030 ***	−0.033 ***
		(−3.54)	(−7.34)	(−7.98)
*GC*		−0.000 *	−0.000 *	−0.000 **
		(−1.91)	(−1.90)	(−2.46)
*WDid*				0.017 **
				(2.44)
*WlnDFD*				0.040 ***
				(4.20)
*WlnPD*				0.037 **
				(2.08)
*WlnEDL*				0.011 *
				(1.84)
*WlnIC*				0.043 ***
				(7.05)
*WGC*				−0.001 **
				(−2.18)
ρ	0.776 ***	0.783 ***		0.765 ***
	(37.40)	(37.60)		(35.52)
λ			0.806 ***	
			(41.72)	
Time FE	Yes	Yes	Yes	Yes
City FE	Yes	Yes	Yes	Yes
$R^{2}$	0.181	0.420	0.340	0.501
Observations	4048	4048	4048	4048

Notes: Z-statistics in parentheses. *** *p* < 0.01, ** *p* < 0.05, * *p* < 0.1. ρ represents the spatial lag term of *GTFEE*; λ represents the spatial lag term of the error term.

**Table 5:** Mechanism analysis of the effect of LCCP on *GTFEE*.

Variables	(5)	(6)	(7)	(8)
	*TL*	*GTFEE*	*TK*	*GTFEE*
*Did*	−0.016 ***	−0.006	−0.135 ***	0.001
	(−4.45)	(−0.38)	(−10.07)	(0.23)
*lnDFD*	−0.043 **	0.004	0.080 ***	−0.005
	(−2.34)	(0.88)	(4.71)	(−1.20)
*lnPD*	0.011	0.015 **	0.077 ***	0.005
	(0.44)	(2.40)	(3.41)	(0.86)
*lnEDL*	0.418 ***	0.001	0.072 ***	−0.022 ***
	(22.02)	(0.21)	(4.06)	(−5.16)
*lnIC*	0.021	−0.032 ***	−0.101 ***	−0.019 ***
	(1.28)	(−7.71)	(−6.70)	(−5.17)
*GC*	0.003 ***	−0.000	−0.001 ***	−0.000
	(11.40)	(−1.32)	(−4.05)	(−0.62)
*WDid*	−0.099 ***	−0.028 ***	−0.125 ***	−0.001
	(−3.66)	(−4.08)	(−4.99)	(−0.18)
*WlnDFD*	0.075 **	0.061 ***	−0.011	0.041 ***
	(2.03)	(6.34)	(−0.33)	(4.93)
*WlnPD*	−0.209 ***	0.017	0.204 ***	0.001
	(−3.02)	(0.94)	(3.14)	(0.06)
*WlnEDL*	−0.320 ***	0.012 *	−0.108 ***	0.030 ***
	(−13.69)	(1.90)	(−4.97)	(5.65)
*WlnIC*	−0.081 ***	0.033 ***	0.097 ***	0.034 ***
	(−3.36)	(5.40)	(4.37)	(6.17)
*WGC*	−0.005 ***	−0.000	0.000	−0.001 ***
	(−4.97)	(−1.38)	(0.15)	(−2.64)
ρ	0.660 ***	0.641 ***	0.708 ***	0.688 ***
	(28.03)	(24.66)	(29.35)	(27.56)
*TL*		−0.076 ***		
		(−8.18)		
*TK*				−0.135 ***
				(−34.83)
Time FE	Yes	Yes	Yes	Yes
City FE	Yes	Yes	Yes	Yes
$R^{2}$	0.107	0.218	0.233	0.347
Observations	4048	4048	4048	4048

Notes: Z-statistics in parentheses. *** *p* < 0.01, ** *p* < 0.05, * *p* < 0.1. ρ represents the spatial lag term of *GTFEE*.

**Table 6:** Balance test results.

Variable	Unmatched (U)	Mean		Bias	|Bias|	*t*-Test Value	*p* > |*t*|
	Matched (M)	Treated	Control	(%)			
*lnPD*	U	5.917	5.828	11.8	82.1	2.77	0.006
	M	5.917	5.901	2.1		0.39	0.696
*lnEDL*	U	10.745	10.071	96.3	95.3	21.29	0.000
	M	10.345	10.714	4.5		1.03	0.304
*lnIC*	U	3.314	3.107	38.2	92.9	9.32	0.000
	M	3.314	3.329	−2.7		−0.56	0.576
*lnDFD*	U	−0.767	−0.820	10.6	76.9	2.61	0.009
	M	−0.767	−0.779	2.4		0.44	0.658
*GC*	U	40.574	37.144	20.9	97.2	6.07	0.000
	M	40.574	40.479	0.6		0.11	0.914

Notes: The control group is the nonpilot cities. The treatment group is the pilot cities. Bias represents the standard deviation of all matching characteristics variables.

**Table 7:** Estimation results of the PSM-SDID Model.

Variables	(9)	(10)	(11)
	SAR	SEM	SDM
*Did*	0.021 ***	0.027 ***	0.031 ***
	(24.13)	(25.50)	(26.45)
*lnDFD*	−0.018 ***	−0.020 ***	−0.019 ***
	(−13.56)	(−14.11)	(−12.46)
*lnPD*	0.005 **	0.008 ***	0.009 ***
	(2.35)	(3.99)	(4.41)
*lnEDL*	0.015 ***	0.020 ***	0.016 ***
	(19.29)	(21.44)	(10.38)
*lnIC*	0.003 ***	0.010 ***	0.012 ***
	(3.51)	(8.45)	(8.80)
*GC*	−0.000 ***	−0.000 **	−0.000 ***
	(−3.35)	(−2.32)	(−2.67)
*WDid*			0.028 ***
			(12.04)
*WlnDFD*			0.006 *
			(1.83)
*WlnPD*			−0.019 ***
			(−3.31)
*WlnEDL*			−0.005 **
			(−2.48)
*WlnIC*			−0.011 ***
			(−5.62)
*WGC*			−0.000
			(−1.39)
ρ	0.410 ***		0.510 ***
	(15.92)		(18.24)
λ		0.584 ***	
		(21.89)	
Time FE	Yes	Yes	Yes
City FE	Yes	Yes	Yes
$R^{2}$	0.459	0.513	0.581
Observations	3538	3538	3538

Notes: Z-statistics in parentheses. *** *p* < 0.01, ** *p* < 0.05, * *p* < 0.1. ρ represents the spatial lag term of *GTFEE*; λ represents the spatial lag term of the error term.

**Table 8:** Estimation results under different spatial weights matrices.

Variables	(12)	(13)	(14)
	W1	W2	W3
*Did*	0.029 ***	0.018 ***	0.009 *
	(7.94)	(4.91)	(1.79)
*lnDFD*	0.019 ***	0.005	0.033 ***
	(3.98)	(1.12)	(4.23)
*lnPD*	0.017 ***	0.015 **	−0.002
	(2.61)	(2.40)	(−0.23)
*lnEDL*	−0.032 ***	−0.013 ***	0.051 ***
	(−6.60)	(−2.73)	(4.48)
*lnIC*	−0.021 ***	−0.033 ***	−0.015 *
	(−5.06)	(−7.88)	(−1.72)
*GC*	−0.000 **	−0.000 **	0.000
	(−2.34)	(−2.42)	(0.44)
*WDid*	0.040 ***	0.016 **	−0.006
	(6.45)	(2.48)	(−0.84)
*WlnDFD*	0.007	0.038 ***	−0.031 ***
	(0.83)	(4.30)	(−3.62)
*WlnPD*	0.043 ***	0.035 **	0.011
	(2.77)	(2.11)	(1.35)
*WlnEDL*	0.041 ***	0.011 *	−0.051 ***
	(7.14)	(1.81)	(−4.43)
*WlnIC*	0.017 ***	0.044 ***	0.015 *
	(3.08)	(7.36)	(1.67)
*WGC*	−0.000	−0.000 *	−0.000
	(−0.22)	(−1.88)	(−0.54)
ρ	0.526 ***	0.710 ***	0.845 ***
	(26.67)	(34.66)	(110.40)
Time FE	Yes	Yes	Yes
City FE	Yes	Yes	Yes
$R^{2}$	0.205	0.201	0.311
Observations	4048	4048	4048

Notes: Z-statistics in parentheses. *** *p* < 0.01, ** *p* < 0.05, * *p* < 0.1. ρ represents the spatial lag term of *GTFEE.* Results are estimated through the SDM method.

**Table 9:** The results of replacing the measurement of the explanatory variable.

Variables	(15)	(16)	(17)
	SAR	SEM	SDM
*Did*	0.044 ***	0.057 ***	0.041 ***
	(8.21)	(5.24)	(9.68)
*lnDFD*	−0.024 **	−0.020 **	−0.019 *
	(−2.36)	(−2.01)	(−1.88)
*lnPD*	0.011 ***	0.020 **	0.015 ***
	(3.52)	(2.19)	(4.78)
*lnEDL*	0.032 *	0.020 **	0.016 ***
	(1.79)	(2.06)	(3.87)
*lnIC*	0.012 ***	0.029 ***	0.024 ***
	(9.16)	(4.75)	(6.58)
*GC*	−0.000 **	−0.000 *	−0.000 *
	(−2.05)	(−1.82)	(−1.67)
*WDid*			0.011 ***
			(9.14)
*WlnDFD*			0.012 ***
			(2.83)
*WlnPD*			0.009 ***
			(4.77)
*WlnEDL*			−0.001 *
			(−1.78)
*WlnIC*			0.008 ***
			(6.23)
*WGC*			−0.002 *
			(−1.89)
ρ	0.479 ***		0.411 **
	(5.25)		(2.45)
λ		0.314 **	
		(2.25)	
Time FE	Yes	Yes	Yes
City FE	Yes	Yes	Yes
$R^{2}$	0.287	0.138	0.311

Notes: Z-statistics in parentheses. *** *p* < 0.01, ** *p* < 0.05, * *p* < 0.1. ρ represents the spatial lag term of *GTFEE*; λ represents the spatial lag term of the error term.

**Table 10:** The results of heterogeneity of urban development levels.

Variables	(18)	(19)	(20)	(21)	(22)
	First	Second	Third	Fourth	Fifth
*Did*	0.031 ***	0.037 ***	0.009 *	0.021	0.001
	(3.46)	(7.18)	(1.83)	(0.23)	(0.13)
*WDid*	0.027 **	0.033 ***	−0.007	−0.062 ***	0.009
	(2.00)	(3.97)	(−0.82)	(−5.59)	(0.49)
ρ	0.603 ***	0.676 ***	0.392 ***	0.372 ***	0.231 ***
	(11.98)	(16.58)	(7.42)	(7.76)	(5.32)
Controls	Yes	Yes	Yes	Yes	Yes
Time FE	Yes	Yes	Yes	Yes	Yes
City FE	Yes	Yes	Yes	Yes	Yes
$R^{2}$	0.650	0.474	0.396	0.430	0.402
Observations	240	464	1088	1280	976

Notes: Z-statistics in parentheses. *** *p* < 0.01, ** *p* < 0.05, * *p* < 0.1. ρ represents the spatial lag term of *GTFEE.* Results are estimated through the SDM method.

**Table 11:** The results of heterogeneity of urban geographic locations.

Variables	(23)	(24)	(25)
	Eastern Cities	Middle Cities	Western Cities
*Did*	0.027 ***	0.019 ***	0.008
	(4.38)	(5.96)	(1.57)
*WDid*	0.033 ***	0.013 **	0.015
	(5.88)	(2.14)	(1.22)
ρ	0.542 ***	0.457 ***	0.221 ***
	(7.98)	(6.18)	(4.27)
Controls	Yes	Yes	Yes
Time FE	Yes	Yes	Yes
City FE	Yes	Yes	Yes
$R^{2}$	0.412	0.386	0.254
Observations	1547	1358	1143

Notes: *t*-statistics in parentheses. *** *p* < 0.01, ** *p* < 0.05. ρ represents the spatial lag term of *GTFEE.* Results are estimated through the SDM method.

## Data Availability

Not applicable.
